# Tomographic evaluation of apexogenesis with human treated dentin matrix in young permanent molars: a split-mouth randomized controlled clinical trial

**DOI:** 10.1186/s12903-025-05997-1

**Published:** 2025-05-07

**Authors:** Nora M. Abo Shanady, Nahed A. Abo Hamila, Gamal M. El Maghraby, Rehab F. Ghouraba

**Affiliations:** 1 Pediatric Dentistry, Preventive Dentistry Department, Faculty of Dentistry, Oral Health, Tanta University, Tanta, 31111 Egypt; 2 Pediatric Dentistry, Oral Health, and Preventive Dentistry Department, Faculty of Dentistry, Tanta University, Tanta, Egypt; 3 Pharmaceutical Technology, Faculty of Pharmacy, Tanta University, Tanta, Egypt; 4 Oral Medicine, Periodontology, Oral Diagnosis and Radiology Department Faculty of Dentistry, Tanta University, Tanta, Egypt

**Keywords:** Apexogenesis, Human treated dentin matrix, Young permanent molars, MTA

## Abstract

**Background:**

The concept of vital pulp therapy (VPT) of immature permanent teeth has evolved in recent years. There has been a great tendency towards investigating new pulp capping materials for perfect imitation for natural dentin-pulp complex formation process and restoring the normal tissue’s characteristics. Therefore, this study aimed to assess the clinical and tomographic outcomes of apexogenesis with human treated dentin matrix (hTDM) compared to mineral trioxide aggregate (MTA).

**Materials and methods:**

40 bilateral deep carious young mandibular first permanent molars (FPMs) in 20 healthy children aged between 6 and 8 years old were randomly allocated into 2 groups in which the FPMs treated with hTDM and MTA after pulpotomy procedure. The children were followed up clinically at 3, 6, 12, and 18 months. Tomographic evaluation was performed at baseline and 18-month evaluation period.

**Results:**

The overall clinical success rate was 100% in both groups. Regarding tomographic evaluation, the mean differences in root length, periapical diameter and area were statistically significant in each individual group but without statistically significant differences between both groups.

**Conclusion:**

Human TDM hydrogel could be considered a promising pulpotomy agent for immature permanent teeth.

**Trial registration:**

The current clinical trial was recorded at clinicaltrials.gov, NCT06116695, 27/10/2023, Retrospectively registered.

## Introduction

Management of cariously exposed immature permanent teeth is one of the challenging clinical situations facing pedodontists compared to mechanical or accidental exposures as their prognosis may be compromised due to the presence of bacteria and their metabolic products that increase the levels of pulpal inflammation [1]. If these carious immature permanent teeth remain untreated, pulp necrosis may occur before the formation of their roots is completed. The development of pulp necrosis in these teeth results in cessation of root development and leaves the tooth with an open apex which often leads to difficult treatment, an unfavorable crown-root ratio, and poor long-term prognosis of the offended tooth [2].Therefore, the treatment should primarily aim to preserve the pulp vitality of such teeth with their rich blood supply and higher cell composition that raise their ability for regeneration and repair giving the teeth the opportunity to complete their root development [3].

Vital pulp therapy (VPT) is considered the best treatment choice for cariously exposed immature permanent teeth [4]. It creates an appropriate environment giving the remaining radicular pulp tissue the opportunity to heal and stimulate different reparative mechanisms for continuation of root formation up to physiological apical closure [5]. It can be accomplished through different procedures such as indirect pulp treatment, direct pulp capping, or pulpotomy procedure depending on the extent of pulp inflammation, perceived stage of bacterial infiltration and damage to the dental pulp. Indirect pulp treatment involves caries removal in 1 or 2 stages using non-selective, selective removal of carious lesion or step wise techniques, leaving thin barrier of sound/affected dentin protecting the underlying pulp tissue. While, direct pulp capping can be performed when a small carious/mechanical pulp exposure, not exceeding 1 mm, is encountered during cavity preparation, or following traumatic injury after controlling hemorrhage [6].

Pulpotomy procedure is one of the most frequently used VPT modalities when the exposed pulp tissue is considered to be affected or damaged. It includes partial and full pulpotomy which differs only in the amount of coronal pulp amputation and the decision depends on hemorrhage control. Partial pulpotomy is performed by amputation of the inflamed pulp tissue 1 to 3 mm or deeper beneath the exposure site to reach healthy pulp tissue. Pulpal bleeding is then controlled by irrigation with a bactericidal agent like sodium hypochlorite and the site of exposure as well as the surrounding dentin are covered with suitable capping agent. When hemostasis is not obtained after a 10-minute exposure to sodium hypochlorite, full pulpotomy can then be performed to the pulpal floor [6, 7].

Mineral trioxide aggregate (MTA) has been recommended as an effective pulp capping agent [6], as a replacement of the traditionally used calcium hydroxide (Ca (OH)_2_), due to its superior antimicrobial properties, sealing ability, marginal adaptation, biocompatibility with adjacent tissues, and thicker dentine bridge formation [8]. However, poor handling, long setting time, tooth discoloration and higher cost have been reported as its potential drawbacks [9]. Therefore, there has been a great demand for discovering an alternative biocompatible, cost-effective pulp capping material that can avoid MTA problems and enhance the natural pulp healing process [10].

Recently, tissue engineering has turned its attention on creating scaffold-cell construct for inducing the regeneration of dental tissue that could mimic the natural process [11, 12]. Human treated dentin matrix (hTDM) has been introduced as a promising pulp capping agent originated from the natural dentin tissue itself that is rich with dentin matrix proteins (DMPs) and growth factors (GFs) [13]. It is a partially demineralized dentin dentin-based biomaterial that is prepared by sequential demineralization of the dentin matrix using some acids as hydrochloric acid, nitric acid, or ethylene diamine tetra acetic acid (EDTA) in certain concentrations and for certain period of time and grinded into small sized particles. It has the same composition of dentin but with different organic/inorganic ratio [14].

In the process of dentin regeneration, hTDM could provide a 3D growth space for the recruited cells simulating the natural extracellular matrix on which dental pulp stem cells (DPSCs) depend for their survival and growth [15]. Demineralization process results in opening the dentinal tubules and liberating the GFs as transforming growth factor β (TGF β), bone morphogenetic proteins (BMPs), fibroblast growth factor(FGF), vascular endothelial growth factor (VEGF), insulin-like growth factor-1(IGF-1) as well as platelet-derived growth factor (PDGF) which are able to naturally stimulate tissue repair and regeneration [16]; these bioactive molecules have the potential to affect the viability, apoptosis and differentiation of DPSCs as they can enhance cellular growth, decline apoptotic markers expression and promote cell survival markers expression. In addition, TDM could also promote mineral synthesis via odontogenic and osteogenic related genes expression providing a good microenvironment for regeneration [17–20].

For dentin-pulp complex regeneration following pulpotomy procedure, there is a great chance for achieving endogenous tissue regeneration through motivating the body’s own biologic resources and reparative capacity to repair/regenerate tissues by using a target-specific biomaterial to recruit endogenous stem cells to the site of injury which could be achieved using hTDM. On that basis, this study aimed to assess the clinical and tomographic outcomes of apexogenesis with hTDM compared to MTA. The null hypothesis (H_0_) postulated that there was no difference in clinical success and tomographic results after immature permanent teeth pulpotomy using hTDM and MTA at all the study evaluation periods.

## Materials & methods

### Study setting and ethical consideration

A split-mouth randomized controlled clinical study was carried out at the Pediatric Dentistry Department Outpatient Clinic, Faculty of Dentistry, Tanta University within the timeframe of January 2022 to October 2023. The hTDM hydrogel preparation was accomplished at the laboratories of the Pharmaceutical Technology Department, Faculty of Pharmacy, Tanta University. The cone beam computed tomography (CBCT) was performed at the Department of Oral Medicine and Periodontology, Oral Diagnosis & Oral Radiology Department, Faculty of Dentistry, Tanta University. The current study adheres to the CONSORT guidelines recommended for randomized controlled trials. The current trial was registered at ClinicalTrials.gov identifier NCT06116695. The approval of the study was accomplished by the ethical committee (REC), Faculty of Dentistry, Tanta University, code (#R- PED 11-21-1) fulfilled the criteria of the Helsinki Declaration of 1964 and its subsequent amendments. Regarding the collected teeth for hTDM material preparation, informed consents from the patients’ parents and ascent from the children were obtained after explaining the purpose of the use of their discarded first permanent premolars for hTDM preparation. Regarding the research candidates, the purpose of the study was explained to their parents and informed consents were obtained before treatment. The researcher was obligated to do dental treatment for every child according to his/her condition as compensation.

### Fabrication of hTDM hydrogel

Dentin matrix was prepared from freshly extracted sound first premolars requiring extraction for orthodontic purposes. The obtained teeth were preserved in sterile phosphate-buffered saline (ReaGene Biosciences, Bengaluru, India) and stored in a refrigerator at 4^o^C immediately after extraction and between the preparation steps. For each tooth, periodontal tissues were completely scraped away using a curette. Enamel, cementum and a part of dentin were completely removed using high speed hand piece with a diamond bur [21]. Access opening was done. The pulp tissues and pre-dentin were then removed using suitable sizes of K-files. The teeth were then cut into smaller blocks to facilitate the penetration of the demineralizing agent using slow speed hand piece with diamond cutting disks and suitable coolant system. The dentin blocks were then immersed in deionized water and placed in an ultrasonic cleaner (Elmasonic S 60 (H), Singen, Germany) for 20 min. The cleaned dentin blocks were treated with 17% EDTA for 10 min, 10% EDTA for 10 min, and 5% EDTA for 5 min to obtain partially demineralized dentin matrix and expose the dentinal tubules. The blocks were then soaked in isopropanol for 2 h to remove any remaining soft tissues in dentin and immersed again in deionized water and placed in the ultrasonic cleaner as before [14]. The resulting hTDM blocks were completely dried and then ground to more smaller particles using the bone crusher (SEDRADENT, Cairo, Egypt) and passed through a set of sieves (Vibarotary sieve, USA) to obtain particles size in the range of 200µ to 450µ. The hTDM powder in sealed bags were exposed to gamma (γ) irradiation Cobalt-60 facility (Mega-gamma-1 type, J 6600 Co-60 Irradiator, Nordion, Canada) at dose of 25 kiloGrays (kGy) for complete sterilization [22]. Hydrogel was prepared using sodium alginate as the principal component. Under complete aseptic conditions, the sterile sodium alginate powder (0.25 g) was dispersed into 4.75 ml of distilled water mixed with 0.25 ml of glycerin as a plasticizer. The sterilized hTDM (0.25 g) was dispersed in the sodium alginate dispersion. Gelation was induced by addition of 0.5 ml of 5% of sterile calcium chloride aqueous solution. The developed hydrogel was loaded into a sterile disposable syringe to obtain a uniform injectable hydrogel mass which was stored in a refrigerator at 4^o^C until required for use [21].

### Eligibility criteria

**Fig. 1:**
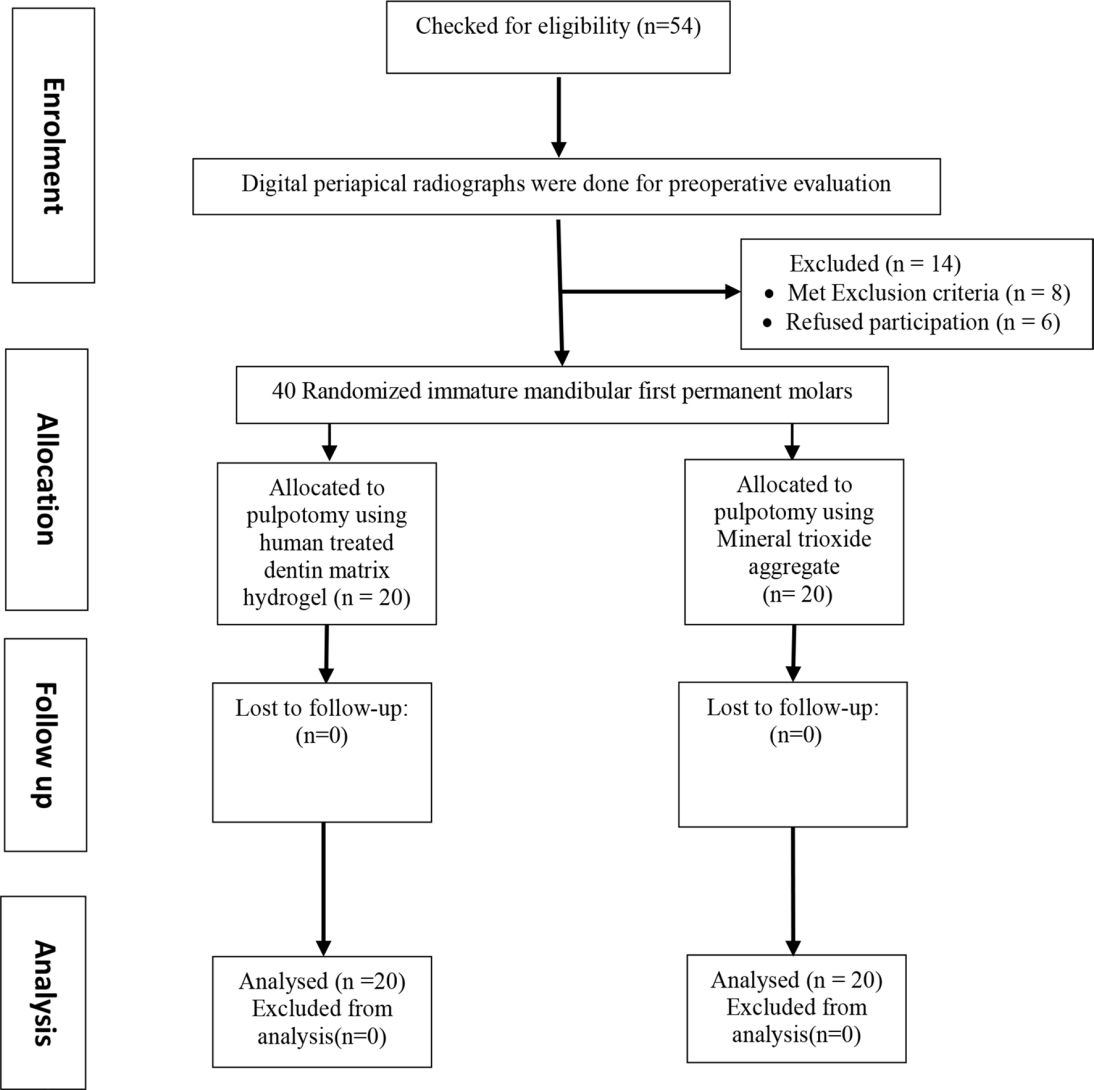
Flowchart entailing the enrollment, allocation, assessment, and sample size analysis of the present study

Inclusion and exclusion criteria, relied on clinical and radiographic evaluation, were used to assess a total number of 54 bilateral mandibular first permanent molars (FPMs) in 27 children aged between 6 and 8 years. The inclusion criteria involved cooperative healthy children having bilateral immature FPMs with deep carious lesions with positive response to pulp testing and normal radiographic appearance. Each child who expressed any clinical signs or symptoms indicating irreversible pulpitis (spontaneous throbbing pain, tenderness to percussion, abnormal tooth mobility, swelling, or sinus tract related to the selected molars), periapical lesion, external or internal root resorption, carious furcation involvement, dystrophic calcification of the pulp, non-restorable tooth, or refused participation was excluded from the current study [23]. Therefore, 14 bilateral mandibular FPMs in 7 patients were excluded leaving 40 bilateral mandibular FPMs in 20 patients which were included in this study. a flow chart that entails enrollment, allocation, assessment, and sample size analysis of the present study was shown in Figure (1). All clinical and radiographic data were recorded in a brief pedodontic examination chart.

### Sample size calculation

Sample size was calculated based on the previous clinical study of Holiel et al.2021 [21] that used hTDM hydrogel in direct pulp capping of permanent mature posterior teeth. Using alpha level = 0.05, effect size d = 0.925 and β level = 0.20 (80% Power); the minimum estimated sample size was 16 teeth per group for a total of 32 teeth. The sample size was increased to 20 teeth per group for a total of 40 teeth to compensate for incomplete results and default rate. The sample size was calculated using G* power computer program version 3.

### Randomization, allocation, and group assignment

Simple randomization was performed using the sealed envelope system. The envelopes were prepared by an individual not included in any phase of the study. Each tooth of the forty chosen molars was given a number and each patient envelope contained two numbers. The prepared envelopes were given to the clinician in the form of opaque sealed envelopes. After the patients met the inclusion criteria and the consents were taken, the selected deep carious molars were randomly allocated in two groups according to the used pulp capping material as the first number selected randomly allocated for group 1 and other number for group 2 as follows:

- **Group-I (experimental group) (*n*** **= 20)**: The FPMs were filled with the prepared hTDM hydrogel.
- **Group-II (control group) (*n*** **= 20)**: The FPMs were filled with white MTA (Pro Root MTA, Tulsa Dental Products, Tulsa, OK, USA).

### Blinding

Owing to the nature of the intervention and the obvious difference between both received treatments, it was not available to blind the operator. However, the research assessors who evaluated the clinical and tomographic conditions as well as the statistical assessor who evaluated the obtained data were blinded.

### Pulpotomy procedure

The pulpotomy procedure was performed by an individual trained operator (first author) who had enough expertise in pediatric endodontic treatment. The procedure started with administration of profound local anesthesia with 2% mepivacaine with 1:20,000 levonordefrin (Alexandria Co., Egypt) and rubber dam isolation (Midwest Dental, Texas, USA) for the selected tooth. Caries was carefully removed using sterile round-carbide bur (no.330) in high-speed handpiece in conjunction with copious water spray and high suction. The remaining soft caries was excavated using sharp spoon excavator and the cavity was inspected for pulp exposure; the exposure size varied between 4 and 5 mm when measured by periodontal probe. Access cavity preparation was performed by deroofing of the pulp chamber using #4 long shank round and/or endo z burs. The coronal pulpal tissue was amputated using sharp spoon excavator up to the canal orifice. The pulp chamber was rinsed with normal saline to remove any debris within the pulp chamber. Normal saline was used instead of sodium hypochlorite due to its high biocompatibility, similar cell osmolarity, and nontoxic effect on DPSCs or GFs. A moistened cotton pellet with sterile saline was then applied with slight pressure for 5 min allowing to achieve complete hemostasis. For group I, freshly mixed hTDM hydrogel was injected on the pulp stump to a thickness of about 3–4 mm. For group II, MTA powder was mixed with the sterile distilled water, according to the manufacturer’s instructions, on a sterile glass slab, the mixture was carried into the pulp stump using an amalgam carrier to a thickness of about 3–4 mm and then it was lightly condensed with a moistened cotton pellet to be gently adapted to the dentinal walls.

After that, in both groups, a small layer of intermediate restorative material (Dentsply DeTrey, Konstanz, Germany) was placed over the pulpotomy agent and the rest of cavity was restored with resin-modified glass ionomer filling material (GC Fuji IX GP EXTRA, GC America Inc., USA). Post-operative digital periapical radiographs were taken immediately to ensure the level of the applied material at the canal orifices, and at 6-month follow-up period to ensure the state of the treated teeth or if there was necessary condition. They were performed using a digital intraoral photo-simulated phosphorus plate sensor (PSP, Planmeca ProSensor HD, Helsinki, Finland) and captured using the XCP extension cone paralleling technique and PSP plate size 2 at the same exposure parameters to ensure standardization (70 kVp, 5 mA and 0.02 s exposure time). All participating cases were recalled after 3, 6, 12 and 18 months for clinical evaluation. The parents were informed about the importance of recall visits to assess the status of the treated permanent molars. They were also instructed to inform the clinician immediately if any pain, discomfort, or swelling was encountered and if the restoration material became deficient or completely removed as the patient would need to receive apexification procedure. Tomographic evaluation was carried out to all the participating children 48 h after treatment (Baseline) and at 18-month follow-up periods.

### Clinical and tomographic evaluation

Two blinded, trained pediatric dentists were involved in clinical and tomographic evaluation. Evaluation of the treated molars was assessed according to the criteria of success reported by the European Society of Endodontology (ESE) [24]. Clinical success criteria included absence of pain (or sensitivity to percussion/palpation), swelling, sinus tract and/or mobility of the treated teeth assessed at the different follow-up periods. Radiographic success criteria involved absence of internal/external root resorption, periapical/inter-radicular radiolucency, irregular calcification, or other pathological changes assessed from the 6-month periapical radiographs & 18-month CBCT images. In addition, continued root development, that is one of the main success criteria, in terms of increasing root length and decreasing apical foramen diameter assessed from baseline and 18-month CBCT images.

The CBCT scanning (KaVo OP 3DVision, Kavo Dental, Biberach, Germany) was performed once each time with the same field of view (8D X 5 H cm) and exposure parameters (120 kV, 5 mA, 0.125 mm voxel size and 7.4 s exposure time) to ensure standardization. Minimal radiation exposure was achieved through choosing the smallest field of view that allowed scanning the FPMs at both sides simultaneously as well as the children were protected using lead aprons. The obtained images were analyzed using the fusion and 3D modules of On Demand 3D software (version 1.0 (build 1.0.10.7462), (× 64 Edition), copyright (c) 2004–2017 Cybermed, and license key 670094709).

**Fig. 2:**
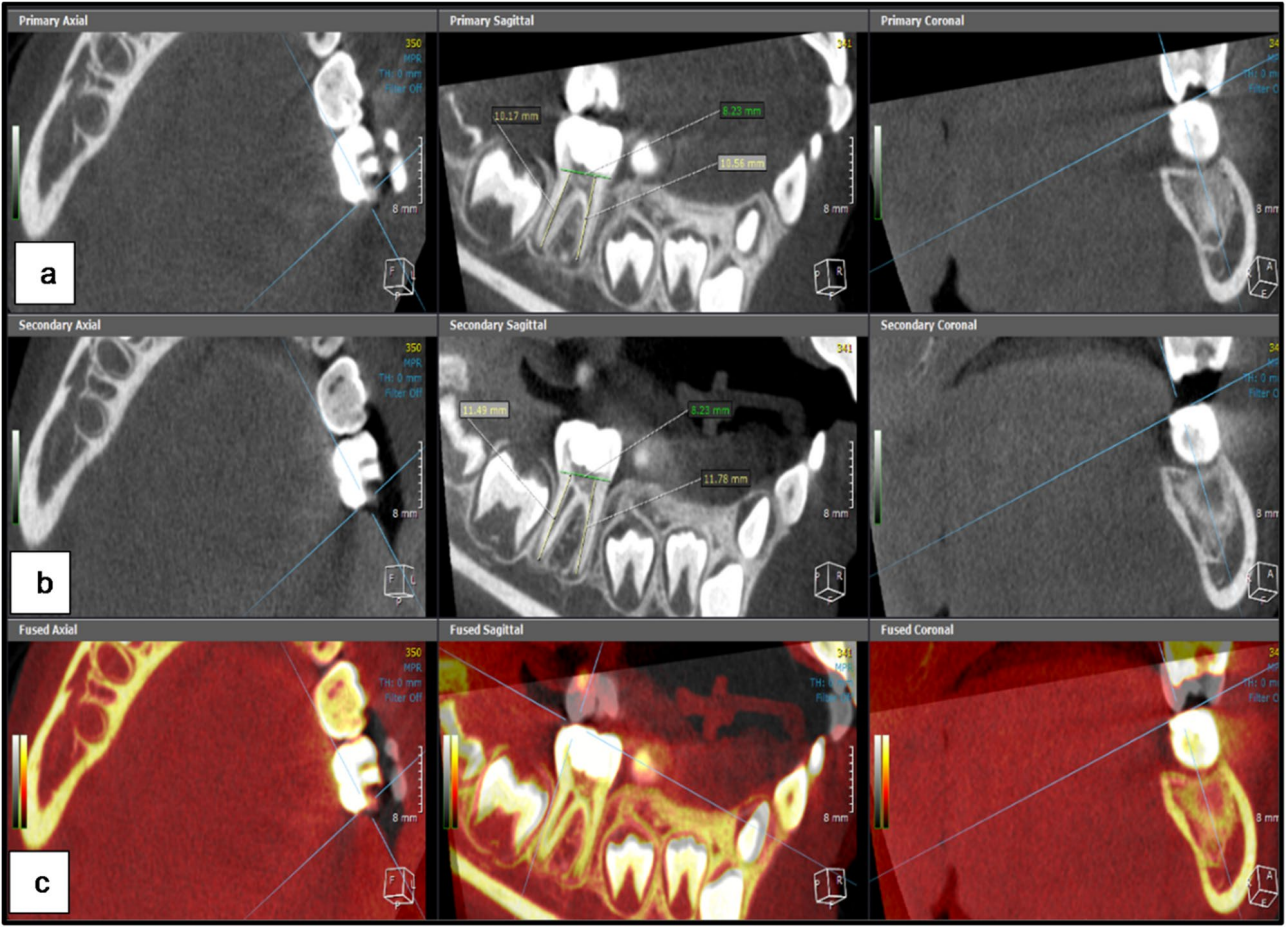
CBCT image shows the measurement of the mesial and distal root length for mandibular left FPM in hTDM group **a**) At the baseline **b**) At18-month follow-up images **c**) Both baseline and eighteen-month CBCT scan superimposed on each other in the same location and direction

**Fig. 3:**
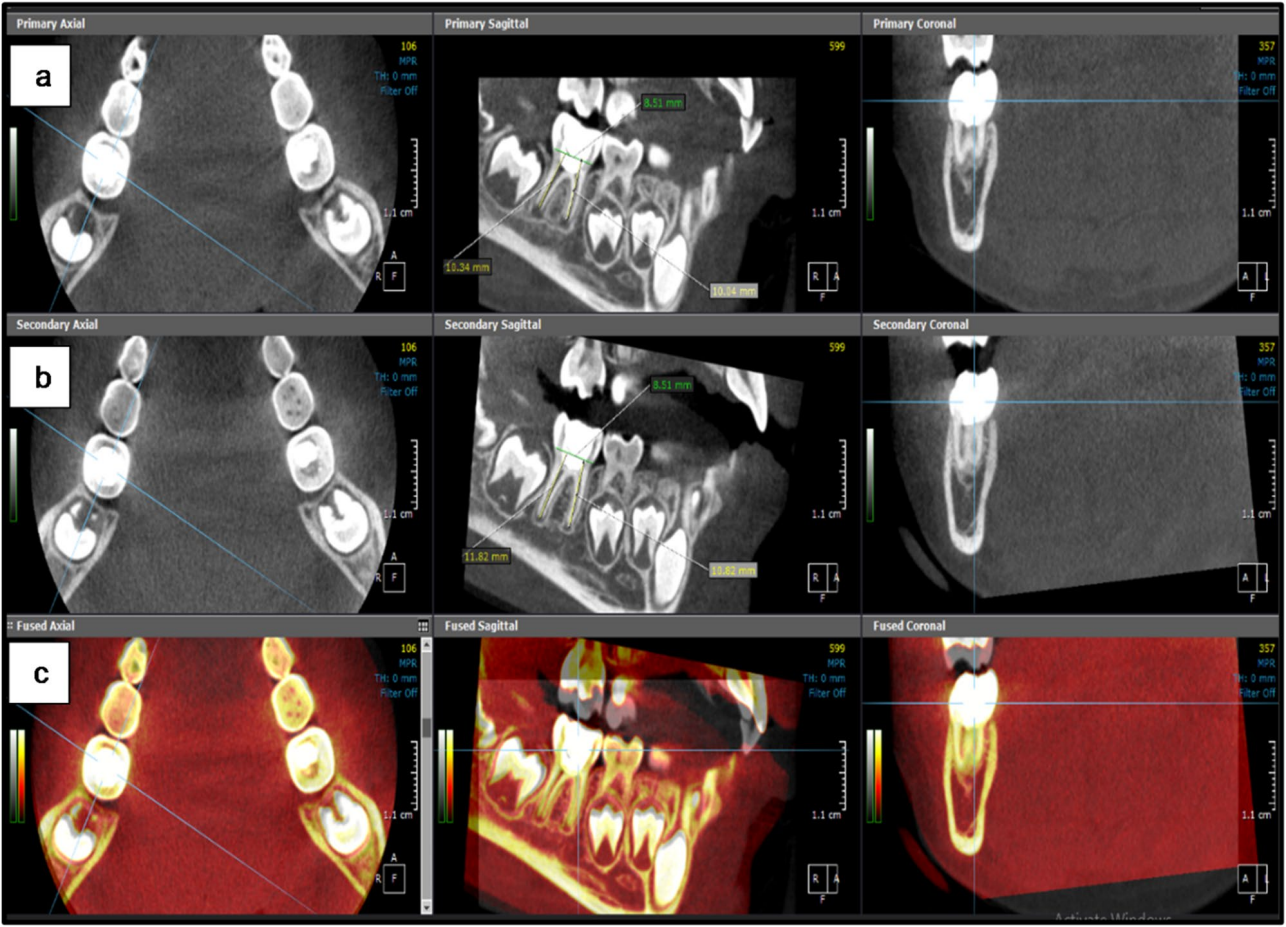
CBCT image shows the measurement of the mesial and distal root length for mandibular right FPM in MTA group **a**) At the baseline **b**) At18-month follow-up images **c**) Both baseline and eighteen-month CBCT scan superimposed on each other in the same location and direction

**Fig. 4:**
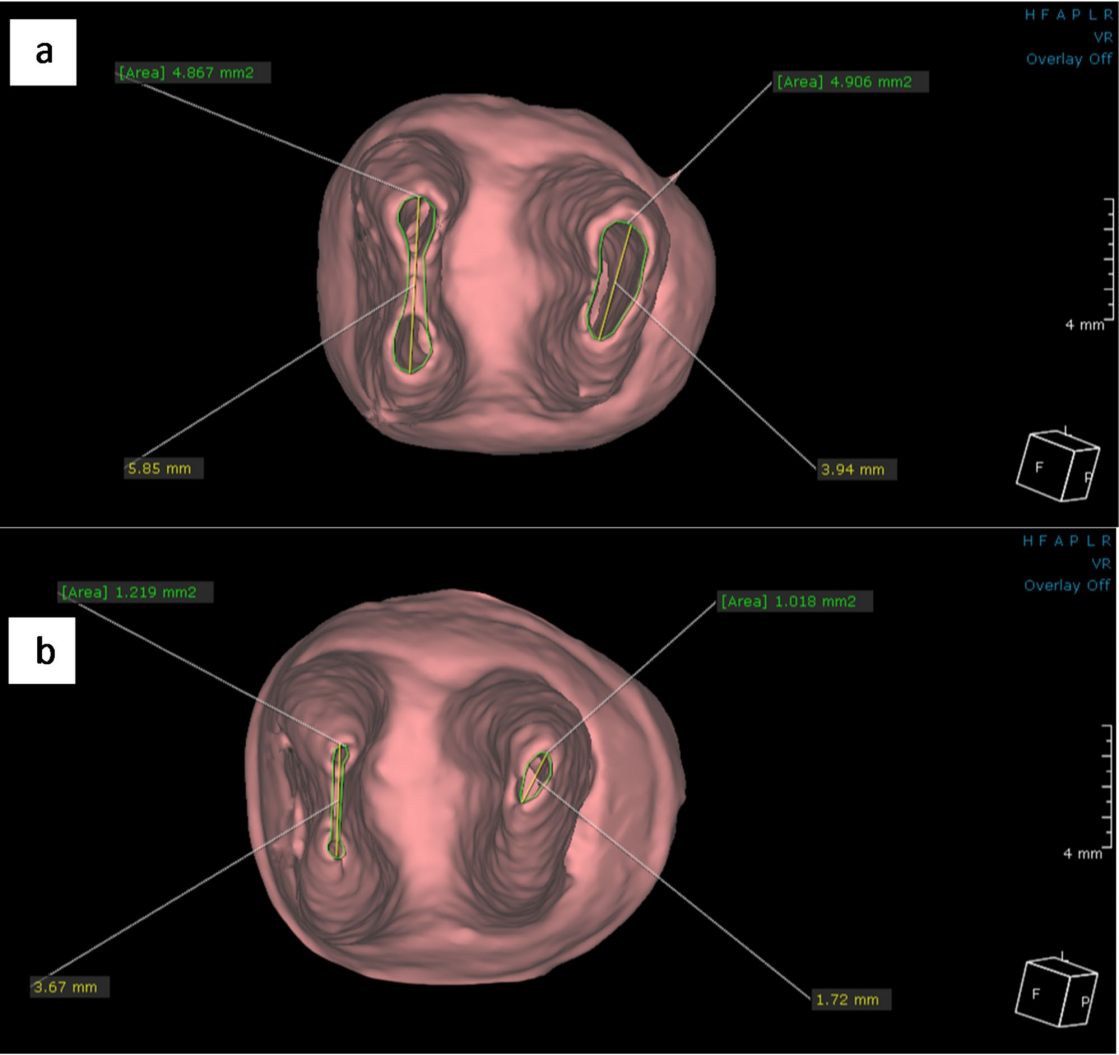
CBCT image shows the measurement of apical foramen length and area in hTDM group **a**) at the baseline **b**)18-month follow-up

**Fig. 5:**
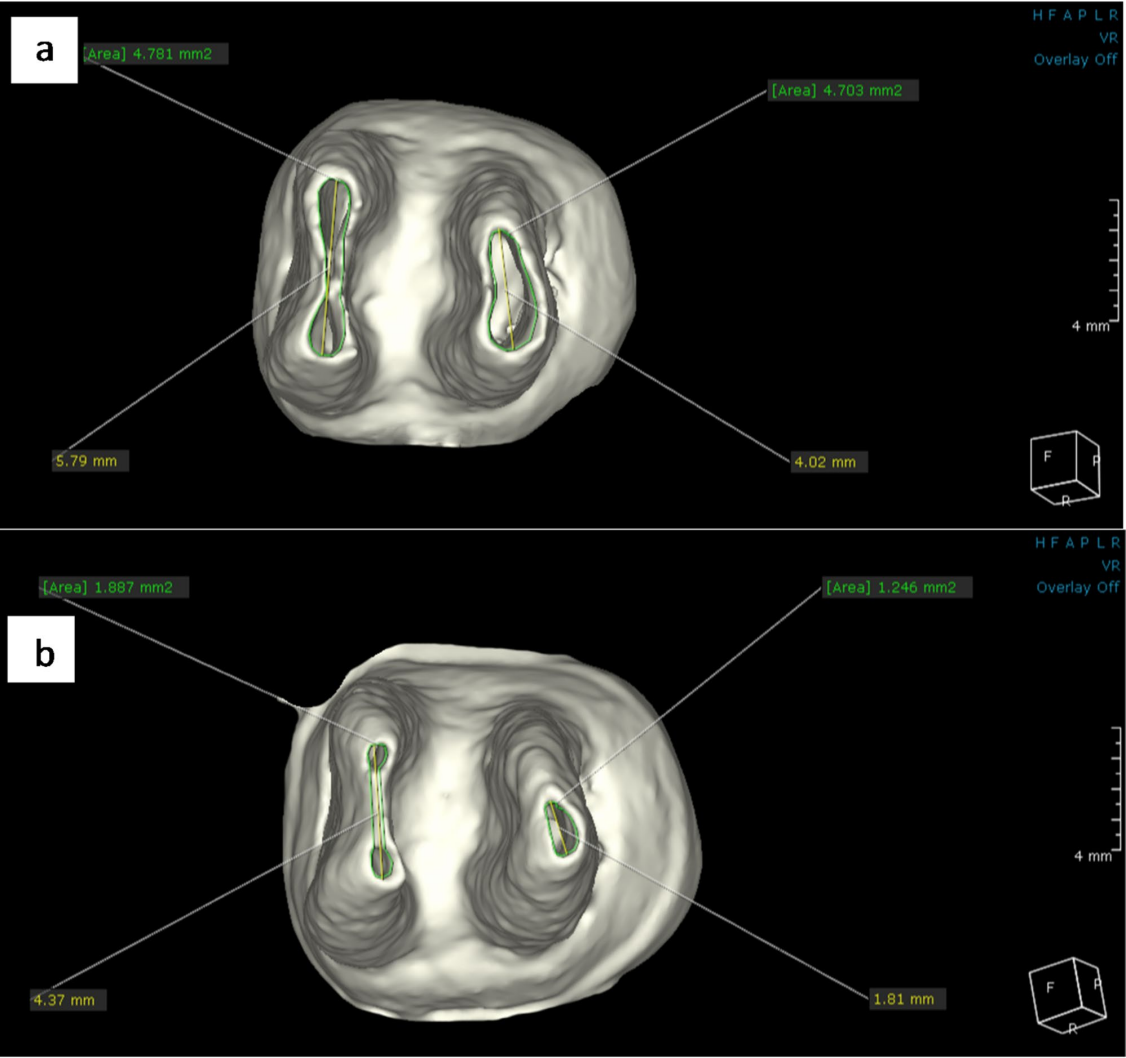
CBCT image shows the measurement of apical foramen length and area in MTA group **a**) at the baseline **b**)18-month follow-up

In the fusion module, the baseline and 18-month follow-up scanning images for each patient were placed in the same section and position to maintain standardization of the FPMs position. The sagittal section was used to evaluate the difference in the root length between both CBCT images for each tooth in both groups. It was performed by determining a line perpendicular on the cemento-enamel junction and then creating a center line through the center of each root extending from this line to the longest point of the root as shown in figures (2–3). Additionally, the 3D module was used to reconstruct STL files of the same mandibular FPMs. The STL files were then imported to the dental module and the 3D reconstructed image was coded with specific color for each group (Pink color for hTDM group and white color for MTA group). Using the apical view, the apical foramen of each root was located, and the apical foramen length and area were measured to evaluate the difference between both CBCT images for each tooth in both groups as shown in figures (4–5).

### Statistical analysis

All data obtained in this study were collected, tabulated and statistically analyzed using SPSS (Statistical Package for the Social Sciences) software package version 26 which developed by IBM, Illinois, Chicago, USA. Qualitative data were presented as frequencies and percentages. The range, mean, and standard deviations of numerical variables were computed. Mann-Whitney U test was used to compare the tomographic changes between the groups and Wilcoxon signed-rank test to study the tomographic changes at the different follow-up periods within each group. The significance level was set at *P* ≤ 0.05.

## Results

All demographic data in terms of age & gender distributions of the participating children were presented in Table 1. A total of 40 pulpotomy procedures were performed in mandibular FPMs in children ranging from 6 to 8 years. All patients were presented for clinical and tomographic follow-up.

**Table 1:** Demographic distribution of study participating children

Demographic characteristics		
Gender	Boys	12 (60%)
	Girls	8 (40%)
Age	Range	6.25–7.42 years
	Mean ± SD	7.025 ± 0.295

### Clinical and radiographic evaluation

At the end of the current study, a considerable improvement in clinical signs and symptoms was revealed in both groups throughout all the follow-up evaluation periods. In both groups, none of the participating cases showed any pain on palpation/percussion, abnormal mobility, or swelling/sinus tract formation related to the FPMs during the different follow-up periods. Additionally, all molars showed radiographic evidence of continued root development with no evidence of pathosis, external/internal root resorption, or irregular calcifications at 6- and 18-month follow-up. All cases were considered clinically and radiographically 100% successful after 18 months.

### Tomographic evaluation

The mean changes in root length of mesial and distal roots were shown in Table 2. When comparing means values of the mesial roots and distal roots of both groups, there was a statically significant increase (*p* ≤ 0.05) regarding intra-group comparison with no statistically significant difference (*p* > 0.05) between the two groups at baseline or after18-month follow-up period.

**Table 2:** Mean changes in root length at baseline and 18-month follow-up

Follow-up	Group	hTDM (*n* = 20)	MTA (*n* = 20)	Z	*P*
Mesial root (mm) Baseline	Mean ± SD	11.01 ± 0.96	11.08 ± 1.18	0.151	0.880
	Median	11.11	11.15		
	Interquartile range	10.32–11.76	10.23–12.17		
18 months	Mean ± SD	12.82 ± 0.84	12.43 ± 0.97	0.680	0.496
	Median	12.96	12.35		
	Interquartile range	11.95–13.52	10.66–13.33		
Z		2.803	2.803		
P		0.005*	0.005*		
Root length mean difference	Mean ± SD	1.81 ± 0.45	1.34 ± 0.61	1.663	0.096
	Median	1.81	1.56		
	Interquartile range	1.38–2.17	0.89–1.66		
Distal root (mm) Baseline	Mean ± SD	11.01 ± 0.87	11.02 ± 1.39	0.076	0.940
	Median	11.15	11.19		
	Interquartile range	10.13–11.87	9.77–11.97		
18 months	Mean ± SD	12.71 ± 0.76	12.42 ± 1.11	1.058	0.290
	Median	12.63	12.28		
	Interquartile range	12.25–13.30	11.53–13.44		
Z		2.803	2.803		
P		0.005*	0.005*		
Root length mean difference	Mean ± SD	1.71 ± 0.58	1.39 ± 0.65	1.361	0.174
	Median	1.78	1.23		
	Interquartile range	1.25–2.02	0.90–1.70		

The mean changes in apical foramen length were presented in Table 3. Upon comparing mean values of apical foramen length regarding mesial and distal roots, it was revealed a statically significant decrease (*p* ≤ 0.05) in apical foramen length among both groups with no statistically significant difference (*p* > 0.05) between the two groups at baseline or after18-month follow-up period. The mean change in apical foramen length was calculated by subtracting the value of the baseline from the value of the 18-month follow-up period. Upon comparing between both groups, there was a statically significant difference (*p* ≤ 0.05) regarding mesial root in favor of hTDM group with no statistically significant difference (*p* > 0.05) regarding distal root apical foramen length change between two groups.

**Table 3:** Mean changes in apical foramen length at baseline and 18-month follow-up

Follow-up	Group	hTDM (*n* = 20)	MTA (*n* = 20)	Z	*P*
Mesial root (mm) Baseline	Mean ± SD	5.13 ± 1.27	5.27 ± 1.33	0.151	0.880
	Median	5.30	5.51		
	Interquartile range	3.87–6.51	4.04–6.25		
18 months	Mean ± SD	3.39 ± 1.02	3.90 ± 1.20	1.058	0.290
	Median	3.51	4.03		
	Interquartile range	2.38–4.27	2.51–4.74		
Z		2.803	2.803		
P		0.005*	0.005*		
Apical foramen length mean difference	Mean ± SD	-1.75 ± 0.52	-1.37 ± 0.52	1.965	0.049*
	Median	-1.86	-1.32		
	Interquartile range	-2.18-(-1.43)	-1.91-(-1.11)		
Distal root (mm) Baseline	Mean ± SD	3.67 ± 1.26	3.64 ± 1.34	0.001	1.000
	Median	4.25	4.25		
	Interquartile range	2.55–4.70	2.54–4.76		
18 months	Mean ± SD	2.27 ± 0.85	2.34 ± 0.96	0.151	0.880
	Median	2.33	2.39		
	Interquartile range	1.59–3.07	1.58–3.28		
Z		2.803	2.803		
P		0.005*	0.005*		
Apical foramen length mean difference	Mean ± SD	-1.41 ± 0.84	-1.31 ± 0.57	0.001	1.000
	Median	-1.37	-1.36		
	Interquartile range	-1.93-(-0.58)	-1.84-(-0.67)		

The mean changes in apical foramen area were presented in Table 4. Upon comparing mean values of apical foramen areas of both roots, there was a statically significant decrease (*p* ≤ 0.05) among both groups with no statically significant difference (*p* > 0.05) between the two groups at base or after 18-month follow-up period. The change in apical foramen area was obtained by subtracting the value of area at the baseline from the value of area after 18-month follow-up period with comparing these changes in apical foramen area between both groups, it was evident, the absence of significant difference (*p* > 0.05) regarding both roots.

**Table 4:** Mean changes in apical foramen area at baseline and 18-month follow-up

Follow-up	Group	hTDM (*n* = 20)	MTA (*n* = 20)	Z	*P*
Mesial root (mm^2^) Baseline	Mean ± SD	5.09 ± 2.17	4.83 ± 1.87	0.227	0.821
	Median	4.90	4.50		
	Interquartile range	2.93–7.13	3.21–6.44		
18 months	Mean ± SD	2.15 ± 1.10	2.64 ± 1.29	1.059	0.290
	Median	2.15	2.40		
	Interquartile range	1.28–2.50	1.61–3.36		
Z		2.803	2.803		
P		0.005*	0.005*		
Apical foramen area mean difference	Mean ± SD	-2.94 ± 1.83	-2.21 ± 1.02	1.058	0.290
	Median	-3.08	-2.56		
	Interquartile range	-3.86-(-1.571)	-3.08-(-1.15)		
Distal root (mm^2^) Baseline	Mean ± SD	4.11 ± 1.87	4.25 ± 2.10	0.378	0.705
	Median	4.56	4.45		
	Interquartile range	2.14–5.36	2.18–5.99		
18 months	Mean ± SD	1.71 ± 0.93	1.87 ± 1.04	0.756	0.450
	Median	1.36	1.65		
	Interquartile range	1.06–2.23	1.21–2.35		
Z		2.803	2.803		
P		0.005*	0.005*		
Apical foramen area mean difference	Mean ± SD	-2.39 ± 1.37	-2.38 ± 1.41	0.529	0.597
	Median	-2.56	-3.08		
	Interquartile range	-3.81-(-0.81)	-3.54-(-0.53)		

## Discussion

Recently, VPT has been directed toward the biomimetic, biological approach based on pulp tissue regeneration capacity for healing and repair [25]. Despite the high success rates of the different available pulp capping agents, they possess limited capability to induce differentiation towards an odontogenic specialization [26]. Dentin matrix, which makes up most of the tooth structure and has bioactive role in natural dentin-pulp complex reparative processes, has been suggested as a suitable scaffold for the regeneration of the pulpal tissues as dentin and pulp are highly correlated tissues. Additionally, dentin matrix might initiate stem cell differentiation and provide the required physical support for cell proliferation and differentiation [27]. TDM is currently considered as an essential component for dental tissue engineering so that this study aimed to assess the outcomes of apexogenesis with hTDM in young permanent molars compared to the currently used MTA.

The present study was focused on immature PFMs in children aged between 6 and 8 years due to the considerable significance of these teeth in maintaining oral and dental health; they are the first permanent teeth to erupt bearing the maximum occlusal forces and they are considered to be the best source of arch anchorage [28]. Loss of these molars at this early age can jeopardize the developing dentition, negatively affecting both arches as well as exposing the child to a traumatic experience from their extraction [29].

The study was designed as a split-mouth with each patient receiving both treatments. The importance of that design is ensuring homogeneity between both groups and eliminating most of the inter-subject variability in terms of age, systemic health, oral hygiene, or caries severity thereby increasing the validity and reliability of the study results. In addition, using the patients as self-control reduces the random error that might take place during inter-patient comparisons maximizing the study’s accuracy and power to detect real differences with fewer participants [30].

The used dentin matrix was stored in phosphate-buffered saline to preserve the dentin biological condition; it is an isotonic, non-fixative, buffer solution (pH = 7.4) with osmolarity and ion concentrations similar to those of the human body so that it can conserve the organic and inorganic components of dentin without affecting its content or permeability [31]. Furthermore, it was treated with different concentrations of EDTA relying on its powerful chelating properties and effective removal of the smear layer exposing the dentinal tubules with their potentially odontogenic factors. The different concentrations of EDTA with their specific incubation times were reported as the optimal treatment method in which the odontogenic proteins and factors are capable of being released [14, 18]. This process was combined with ultrasonic cleaning aiming to improve the smear layer removal as well as loosen the inter-tubular and peritubular dentin structure [32].

The particle size used in the present study was in the range of 200µ to 450µ which coincides with the reported particle size of the TDM used in dentin-pulp regeneration (ranged between 76 µ [18] to 500 µ [21]). This is intended to ensure better accommodation of the material to the defect side, maintaining larger dentin particles that have better regenerative potential as well as avoiding the smaller dentine particles that have higher resorbability prior to hard tissue formation. The prepared hTDM was sterilized using Gamma radiation which differs from the earliest studies using hTDM as Li et al. 2011 [14], Na et al. 2016 [17], Chen et al. 2017 [18], and Holiel et al.2021 [21] that relied on soaking it in phosphate-buffered saline with penicillin and streptomycin for 72 h to achieve the purpose of the material disinfection but, it still had a risk of infection. Gamma radiation sterilization has greater effectiveness, higher certainty of sterility and deeper penetration. Additionally, it doesn’t need any chemicals or heat dependence so that it can preserve the integrity of the growth factors [33, 34].

In the current study, the pulpotomy procedure was evaluated both radiographically and tomographically. Radiographic evaluation was done twice at 6 months to identify the possible abnormal changes that could take place, and also to check the evidence of continued root development and the integrity of the placed restoration [35]. Digital radiographs were taken with as low exposure parameters as possible (70 kVp, 5 mA), shorter exposure time (0.02 s), and using rectangular collimation to significantly reduce the radiation dose (1 µSv per exposure) [36]. Accordingly, it represents only an extremely small portion of extra radiation for a child. On the other hand, tomographic evaluation was done to overcome the limitations of 2D radiographs in accurate measuring of the changes that takes place in the developing roots and the apical foramen [37]. The CBCT performed using the smallest field of view that can accommodate the region of interest (8D X 5 H cm) eliminating the need for exposure of the child to extra radiation. Also, appropriate exposure parameters (120 kV, 5 mA, 0.125 mm voxel size and 7.4 s exposure time) allow accurate observation and measurements. The effective dose that the child exposed to using these medium exposure parameters was reported to be about 177 µSv which is much higher compared with periapical radiographs [37]. However, each participating child received CBCT examination only 2 times within eighteen months interval and was protected using lead aprons.

In the current study, the overall clinical and radiographic success rate of hTDM and MTA after 18 months was 100%. These results were in accordance with Mehrvarzfar et al.2018 [19] who performed partial pulpotomies on bilateral third molars in 11 healthy volunteers using MTA alone and a combination of MTA/TDM in a split mouth manner and they found that both groups were similar in both clinical and radiographic parameters after 6-week clinical and radiologic assessment. The results also agreed with Holiel et al. 2021 [21] who applied direct pulp capping using hTDM, MTA, and Biodentine to 45 traumatically exposed molars distributed equally into 3 groups and reported that all cases were clinically and radiographically successful after 2 years follow-up.

Regarding MTA clinical success rates, they agreed with El-Meligy and Avery 2006 [38] (100%), Ghoddusi et al. 2012 [39] (100%), Nosrat et al. 2013 [23] (100%), Keswani et al.2014 [11](100%), Eppa et al. 2018 [5] (100%), Vafaei et al.2022 [40] (100%), and Mousivand et al.2022 [41] (95%) who compared MTA with different pulp capping materials such as Ca (OH)_2_, zinc oxide eugenol, calcium enriched mixture, platelet rich fibrin, triple antibiotic paste, abscess remedy following partial/full pulpotomy in immature permanent molars. On the other hand, the radiographic success rates of majority of these studies didn’t match the same results in the present study as Ghoddusi et al. 2012 [39] (75%), Nosrat et al. 2013 [23] (81.5%), Keswani et al.2014 [11]( 80.07%), Vafaei et al.2022 [40] (84.5%). Additionally, clinical and radiographic results weren’t in accordance with Peng et al.2015 [42] (91%, 65%) and Abuelniel et al.2021 [43] (80%, 70%). This disagreement is probably due to the use of different types of MTA in these studies as well as different teeth samples with different pulp conditions were evaluated (some with irreversible pulpitis or traumatic pulp exposures).

In the current study, both materials showed great ability to trigger continuous root development of immature FPMs with statistically significant differences between baseline and 18-month evaluation period. The mean differences in root length, apical foramen length and area in MTA group were 1.34 mm, -1.37 mm, -2.21 mm^2^ regarding the mesial root and1.39 mm, -1.31 mm, -2.38 mm^2^ regarding the distal root. These results agreed with Vu et al.2020 [44] who reported 1.286 mm and − 1.66 mm^2^ mean differences in root length and apical foramen area in MTA partial pulpotomy performed on 50 immature first permanent premolars with carious or traumatic pulp exposures after 12 months.

Additionally, these mean differences in hTDM group were (1.81 mm, -1.75 mm, -2.94 mm^2^ regarding the mesial root and 1.71 mm, -1.75 mm, -2.39 mm^2^ regarding the distal root) without statistically significant differences with MTA group except for mesial apical foramen length. Despite the lack of studies evaluating hTDM in immature permanent teeth, the results of the current studies ensure the regenerative capacity of hTDM material and its ability to express the bioactive molecules needed for VPT. This ability was confirmed by Chun et al.2011 [45] who analyezed, in their in-vitro study, the soluble DMPs released from TDM and found as much as 147 kinds of bioactive molecules varied between GFs, enzymes, signaling molecules, and transcription factors. Another study by Avery et al.2017 [46] who investigated the amount of the liberated GFs from the dentin matrix treated with identified that TGF-1 was demonstrated with the highest concentration followed by BMPs, VEGF, FGF-2, PDGF, IGF-1, BMP-4, and BMP-7. They also reported that these GFs were present at physiological levels with no toxicity issues and no potential for ectopic mineral deposition.

Further, the regenerative capacity of hTDM is also confirmed in some histological studies like Na et al. 2016 [47]who seeded stem cells inside the pulp space of the prepared TDM blocks, implanted them on the back of immunodeficient mice, and examined the mice histologically after 6 weeks to find that the pulp cavity was filled with pulp-like and vascular-like tissue with continuous dentin-like tissue of uniform thickness deposited on the surface of hTDM blocks. Other studies by Mehrvarzfar et al.2018 [19]and Holiel et al. 2021 [20]confirmed thicker dentin bridge formation with more homogenous tubular dentin and less inflammatory cell infiltrates in hTDM groups compared to the other studied pulp capping materials ensuring its good potential to regenerate dentin as well as promoting regeneration of pulp tissue.

### Clinical relevance

The disadvantages of the traditional MTA in VPT of immature permanent teeth could be avoided by searching for new biomimetic, bioactive, cost-effective materials such as hTDM hydrogel material which reveal comparable clinical and radiographic results to MTA.

## Conclusion

Vital pulp therapy of immature permanent teeth with carious exposure is considered mandatory to allow continuous root development to withstand the force of mastication. With the presence of several materials that have been used in VPT, which may be costly or unavailable, urging the need for alternative materials easy to obtain at any time with sufficient regenerative properties. This could be fulfilled using hTDM material with multitude of GFs and other bioactive molecules revealed by 100% successful apexogenesis confirmed by comparable clinical and radiographic results with MTA in the treatment of cariously exposed immature permanent teeth.

## Limitations

The present study had some limitations regarding the prepared hTDM hydrogel material; many preparation steps were needed to reach the required hTDM powder prior to its use. In addition, the need for preparing the TDM powder every 6 months and fresh mixing of hydrogel components each time prior its application results in discarding the excess and wasting the remaining material. This necessitates searching for the capability of preserving the material bioactivity for longer time. Although these steps may appear costly, the amount of the dentin matrix hydrogel obtained in a single process is considered huge enough for several cases with the same cost in comparison to the amount of MTA needed for a single case.

## Data Availability

On reasonable request, the datasets utilized and/or analyzed during the present study are accessible from the corresponding author.
